# Capillary leak syndrome during continuous renal replacement therapy after renal hilum ligation in a hypercapnic landrace pig

**DOI:** 10.5935/2965-2774.20230139-en

**Published:** 2023

**Authors:** Yuri de Albuquerque Pessoa dos Santos, Luis Carlos Maia Cardozo Júnior, Pedro Vitale Mendes, Bruno Adler Maccagnan Pinheiro Besen, Marcelo Park

**Affiliations:** 1 Intensive Care Unit, Hospital das Clínicas, Faculdade de Medicina, Universidade de São Paulo - São Paulo (SP), Brazil

Literature on dialysis in pigs is scarce, and there is no description of capillary leak syndrome during dialysis in pigs. Our aim in this paper is to bring attention to the possible occurrence of this syndrome, at least in the described specific condition.

In an ongoing experimental line of research, we are investigating the impact of high bicarbonate concentrations on blood pH during continuous venous-venous hemodialysis (CVVHD) in hypercapnic pigs with severe renal failure induced by hilum ligation.^(1)^ In this described experiment, a female 31kg Landrace pig was used as the research subject. After anesthesia, a central venous line, venous dialysis catheter (12-French, 16cm, Arrow^TM^, PA, USA), Swan-Ganz catheter (Edwards Lifesciences^TM^, Irvine, USA), and an arterial line were inserted. After a median laparotomy, a cystostomy was performed (to ascertain anuria), and the renal hilum was ligated en bloc. The pig was stabilized for one hour after the end of the surgery. Then, the tidal volume was reduced to 2/3 (Table 1), and after one hour of tidal volume reduction, CVVHD was started.

**Table 1:** Hemodynamic, respiratory and metabolic variables of the subject during the experiment

	Baseline	15 minutes	30 minutes	45 minutes	CVVHD	15 minutes	30 minutes	1 hour	2 hours	3 hours	4 hours
Hemodynamic											
Heart rate (bpm)	85	94	122	109	111	94	99	119	153	211	220
ABPm (mmHg)	90	92	116	88	89	56	69	78	61	64	47
CVP (mmHg)	8	8	6	6	7	6	5	5	4	2	3
PAOP (mmHg)	10	12	10	10	9	10	6	5	4	13	17
PAPm (mmHg)	25	27	29	29	29	23	23	23	27	26	17
Lactate (mEq/L)	1,0				0.6				0.6	0.7	2.2
Norepinephrine (mcg/kg/minute)	0	0	0	0	0	0	0	0.1	0.2	0.3	1.2
Respiratory											
Tidal volume (mL)	240	160	160	160	160	160	160	160	160	200	240
Respiratory rate (IPM)	40	40	40	40	40	40	40	40	40	40	40
PEEP (cmH_2_O)	5	5	5	5	5	5	5	5	5	5	5
FiO_2_ (%)	21	40	40	40	40	40	40	30	30	25	25
PaO_2_ (mmHg)	71				101				75	73	83
PaCO_2_ (mmHg)	43				80				56	42	33
HCO_3_ (mEq/L)	22.7				23.7				20.3	17.6	14.8
SBE (mEq/L)	-2.2				-4.8				-6.8	-8.4	-10.8
SatO_2_ (%)	93				95				94	88	93
Metabolic											
Core temperature (°C)	36.3	37.2	37.5	37.6	37.5	36.1	36.1	36.2	36.4	36.3	36.8
pH	7.34				7.09				7.17	7.23	7.26
Glicemia (mg/dL)	102				116				130	127	283
Cai^+^2^^ (mMol/L)	1.31				1.39				1.31	1.32	1.36
K^+^ (mEq/L)	5				5.2				5.8	5.6	6.4
Na^+^ (mEq/L)	136				137				135	137	134
Cl^-^ (mEq/L)	112				111				113	117	116
Blood flow (mL/minute)	0	0	0	0	0	205	207	209	212	211	209
Net ultrafiltration (mL)	0	0	0	0	0	11	26	53	74	87	143
Fluid intake^*^ (mL)	0	0	0	0	0	0	250	250	500	800	1300
Fluid balance (mL)	0	0	0	0	0	-11	224	197	426	713	1157

CVVHD - continuous venous-venous hemodialysis; bpm - beats per minute; ABPm - mean systemic arterial blood pressure; CVP - central venous pressure; PAOP - pulmonary artery occlusion pressure; PAPm - mean pulmonary artery pressure; IPM - inspirations per minute; PEEP - positive end expiratory pressure; FiO_2_ - fraction of inspired oxygen; PaO_2_ - partial pressure of oxygen; PaCO_2_ - partial pressure of carbon dioxide; HCO_3_ - bicarbonate. SBE - base excess; SatO_2_ - oxygen saturation. Standard base excess and mcg/kg/minute denotes micrograms per kg per minute. From baseline to continuous venous-venous hemodialysis initiation, tidal volume was reduced to 2/3 of the baseline.

* Normal saline was used for fluid administration.

**Figure 1:**
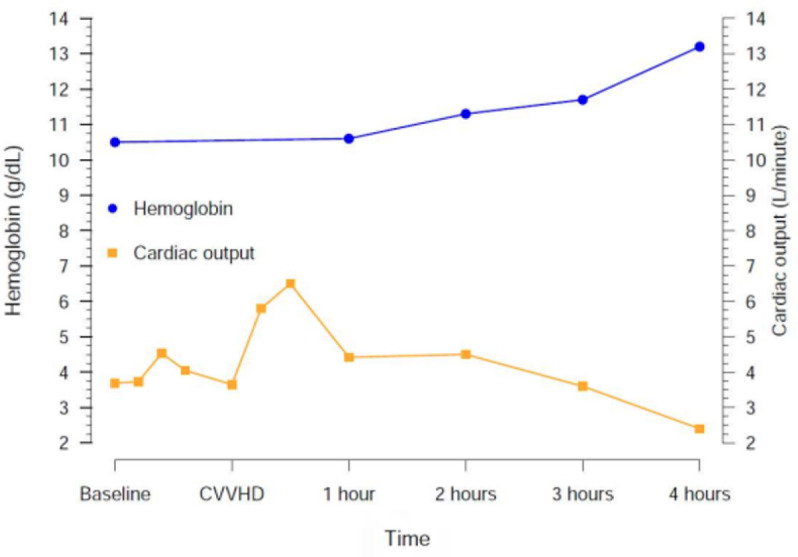
Change in hemoglobin and cardiac output during the five hours of the experiment.

In the first experiments, CVVHD was performed using the Fresenius F8® (Fresenius Medical Care^TM^, MA, USA) low-flow filter without the occurrence of any adverse events or complications.^(1)^ Due to a shortage of this filter in the Brazilian market, we changed to an Elisio-H17® filter (Nipro Medical LTDA^TM^, Sorocaba, São Paulo, Brazil). Surprisingly, in the first experiment using the new filter, fatal refractory shock, low cardiac output, and hemoconcentration occurred soon after continuous renal replacement therapy initiation. Figure 1 shows the change in hemoglobin and cardiac output during stabilization, hypoventilation and four hours of CVVHD. Table 1 shows the temporal evolution of other hemodynamic, respiratory, and metabolic variables during the experiment. Hemoconcentration and the dramatic fall in cardiac output occurred at the same time as the subject exhibited severe tachycardia, high norepinephrine levels, and a need for normal saline. We also observed a decrease in central venous pressure and mean pulmonary artery pressure despite a terminal rise in pulmonary artery occlusion pressure. The terminal paradoxical behavior of the low mean pulmonary artery pressure and high pulmonary artery occlusion pressure could be explained by the association of hypovolemia with left ventricle dysfunction.

The difference between baseline levels and levels after continuous venous-venous hemodialysis initiation and cardiac output was measured every 15 minutes. After continuous venous-venous hemodialysis initiation, cardiac output was measured after 15 and 30 minutes and every hour thereafter. CVVHD - continuous venous-venous hemodialysis.

The hemoconcentration with the associated severe hypovolemic hemodynamic behavior in this case is very similar to the capillary leak syndrome as described by Dr. Bayard Clarkson, which may occur with extracorporeal support systems.^(2-4)^ Many pathophysiological pathways are linked to capillary leak syndrome, which is a condition that is associated with a high mortality.^(2)^ In advanced phases, severe myocardial depression can occur, similar to what occurred in our experiment.^(5)^ In later experiments, we tried additional filters, such as Diacap Acute L® (BBraun^TM^, São Gonçalo, Rio de Janeiro, Brazil), Poliflux® (GambroTM, São Paulo, Brazil), and Fx80® (Fresenius Kabi Brazil LTDA^TM^, Itapecerica da Serra, São Paulo, Brazil), without any systemic reaction of the subject.

This paper demonstrates that capillary leak syndrome may occur in experiments that are conducted using extracorporeal systems. This phenomenon is something that translational researchers should be aware of to help with the appropriate choice of dialyzers in pigs.
